# Subtype-specific overexpression of the Rac-GEF P-REX1 in breast cancer is associated with promoter hypomethylation

**DOI:** 10.1186/s13058-014-0441-7

**Published:** 2014-09-24

**Authors:** Laura Barrio-Real, Lorena G Benedetti, Nora Engel, Yaping Tu, Soonweng Cho, Saraswati Sukumar, Marcelo G Kazanietz

**Affiliations:** 1 Department of Pharmacology, Perelman School of Medicine, University of Pennsylvania, 1256 Biomedical Research Building II/III, 421 Curie Boulevard, Philadelphia, 19104 PA USA; 2 Department of Biochemistry, Fels Institute for Cancer Research and Molecular Biology, Temple University, 3307 North Broad Street, Philadelphia, 19140 PA USA; 3 Department of Pharmacology, Creighton University, 2500 California Plaza, Omaha, 68178 NE USA; 4 Department of Oncology, Johns Hopkins University School of Medicine, 2024 East Monument Street, Baltimore, 21231 MD USA

## Abstract

**Introduction:**

The Rac-GEF P-REX1 is a key mediator of ErbB signaling in breast cancer recently implicated in mammary tumorigenesis and metastatic dissemination. Although P-REX1 is essentially undetectable in normal human mammary epithelial tissue, this Rac-GEF is markedly upregulated in human breast carcinomas, particularly of the luminal subtype. The mechanisms underlying P-REX1 upregulation in breast cancer are unknown. Toward the goal of dissecting the mechanistic basis of P-REX1 overexpression in breast cancer, in this study we focused on the analysis of methylation of the *PREX1* gene promoter.

**Methods:**

To determine the methylation status of the *PREX1* promoter region, we used bisulfite genomic sequencing and pyrosequencing approaches. Re-expression studies in cell lines were carried out by treatment of breast cancer cells with the demethylating agent 5-aza-2′-deoxycitidine. *PREX1* gene methylation in different human breast cancer subtypes was analyzed from the TCGA database.

**Results:**

We found that the human *PREX1* gene promoter has a CpG island located between -1.2 kb and +1.4 kb, and that DNA methylation in this region inversely correlates with P-REX1 expression in human breast cancer cell lines. A comprehensive analysis of human breast cancer cell lines and tumors revealed significant hypomethylation of the *PREX1* promoter in ER-positive, luminal subtype, whereas hypermethylation occurs in basal-like breast cancer. Treatment of normal MCF-10A or basal-like cancer cells, MDA-MB-231 with the demethylating agent 5-aza-2′-deoxycitidine in combination with the histone deacetylase inhibitor trichostatin A restores P-REX1 levels to those observed in luminal breast cancer cell lines, suggesting that aberrant expression of P-REX1 in luminal breast cancer is a consequence of *PREX1* promoter demethylation. Unlike *PREX1*, the pro-metastatic Rho/Rac-GEF, *VAV3*, is not regulated by methylation. Notably, *PREX1* gene promoter hypomethylation is a prognostic marker of poor patient survival.

**Conclusions:**

Our study identified for the first time gene promoter hypomethylation as a distinctive subtype-specific mechanism for controlling the expression of a key regulator of Rac-mediated motility and metastasis in breast cancer.

**Electronic supplementary material:**

The online version of this article (doi:10.1186/s13058-014-0441-7) contains supplementary material, which is available to authorized users.

## Introduction

Rho/Rac GTPases are effectors of cell surface receptors that control fundamental cellular functions, including actin cytoskeleton dynamics, cell morphology, motility, and the progression through the cell cycle [1]. Like most GTPases, Rac cycles between a GDP-bound inactive state and a GTP-bound active state responsible for the activation of downstream effectors. This switch is tightly regulated by Rac guanine nucleotide exchange factors (Rac-GEFs) that promote GTP loading onto Rac, and Rac GTPase activating proteins (Rac-GAPs) that inactivate Rac by accelerating GTP hydrolysis [1]-[3]. Extensive evidence supports a role for Rac in tumorigenesis as well as in the acquisition of a highly motile phenotype required for metastatic dissemination of cancer cells [4]-[6]. Alterations of Rac signaling are common in human cancer and can involve upregulation of Rac itself, expression of an active spliced variant (Rac1b), or very rarely Rac gain-of-function mutations and Rac-GAP downregulation [7]-[13]. However, the most common mechanism that accounts for Rac hyperactivation in human cancer is the dysregulation of Rac-GEF function [14]-[16]. A number of studies have indeed reported overexpression and activating mutations of Rac-GEFs in cancer, as described for Tiam1, Trio, and others [14]-[21]. Exacerbated inputs from pathways required for the activation of Rac-GEFs, such as PI3K or receptors that are coupled to PI3K activation (for example HER2/ErbB2 or PDGF receptors), also confer Rac hyperactivation [3],[6],[22],[23].

Using a PCR-based array screening approach, our laboratory previously reported that the PI3K- and Gβγ-dependent Rac-GEF P-REX1 is highly expressed in breast cancer [24]. P-REX1 was found to be an essential mediator of HER2-driven activation of Rac and motility in breast cancer cells by integrating signals emanating from tyrosine-kinases and G-protein-coupled receptors (GPCRs) [24],[25]. RNA interference (RNAi)-mediated silencing of P-REX1 essentially impairs the ability of breast cancer cells to form tumors in nude mice as well as their migratory capacity, suggesting its potential involvement in breast tumorigenesis and metastasis [24]. P-REX1 is essentially undetectable by immunohistochemistry (IHC) analysis in human normal mammary epithelial tissue, whereas its expression can be readily detected in approximately 60% of breast tumors [24]. Elevated P-REX1 expression in breast tumors has been associated with poor outcome and development of metastasis in patients [24],[25]. Analysis of P-REX1 expression using the Netherlands Cancer Institute (NKI) microarray data and more recently through metagenomic analysis of Rho/Rac GEFs established that P-REX1 upregulation occurs in a subset of tumors, specifically those of the luminal subtype. On the other hand, P-REX1 levels are low in basal-like breast cancer, a subtype with high abundance of triple-negative (estrogen receptor (ER)-, progesterone receptor (PR)-, and human epidermal growth factor receptor (HER2)-negative) tumors [21],[24]. In addition to P-REX1, luminal tumors express high levels of VAV3, a Rho/Rac GEF that was found to drive a lung-specific metastatic transcriptional program in breast cancer cells [21]. Consistent with data observed in breast cancer, P-REX1 has been implicated in metastasis in prostate cancer and melanoma [26],[27].

As luminal breast cancer is the most common subtype and responsible for the largest number of breast cancer deaths [28],[29], understanding the regulation and function of these Rac-GEFs is highly relevant. Deciphering the mechanisms leading to overexpression of tumorigenic and metastatic proteins is key to identify novel approaches to counterbalance dysregulated oncogenic stimuli in cancer. In this regard, the molecular mechanisms underlying P-REX1 upregulation in luminal breast cancer remain unknown. Extensive evidence suggests that epigenetic events including DNA methylation and histone modifications play important roles in the transcriptional regulation of oncogenic/metastatic genes in many cancer types, including breast cancer [30]-[32]. DNA methylation of promoter CpG islands results in transcriptional silencing, and dysregulation of this epigenetic mechanism plays an important role in oncogenesis and cancer progression [31]. Towards the goal of dissecting the mechanistic basis of P-REX1 overexpression in breast cancer, in this study we focused on the analysis of methylation of the *PREX1* gene promoter. We found that derepression of P-REX1 expression in luminal breast cancer involves the demethylation of its promoter. A comprehensive analysis of human mammary cell lines and patient-derived tumors revealed marked differences in *PREX1* promoter methylation in distinct breast cancer subtypes that inversely correlate with P-REX1 expression levels. The dissection of the mechanisms leading to P-REX1 upregulation in breast cancer may have significant prognostic and therapeutic value.

## Methods

### Cell lines

Human breast cell lines (BT-474, BT-549, HCC1419, HMEC, MCF-10A, MCF-7, MDA-MB-231, MDA-MB-361, MDA-MB-453, MDA-MB-468 and T-47D) were obtained from the American Type Culture Collection (ATCC; Manassas, VA, USA), except for HMEC cells that were purchased from Lonza (Walkersville, MD, USA). Cells were grown in the medium recommended by the providers.

### Western blot analysis

Western blot analysis was carried out essentially as described previously [33]. Briefly, cells growing in monolayer at a confluence of 80% were lysed in RIPA buffer (50 mM Tris-HCl, 150 mM NaCl, 1 mM EDTA, 1% NP-40, 0.5% sodium deoxychloride, and protease/phosphatase inhibitors). Ten μg of total protein lysate were loaded onto 8% acrylamide gels, transferred into PVDF membranes, and incubated with anti-human P-REX1 antibody (1:1,000, Sigma-Aldrich; St. Louis, MO, USA) or anti-ER-alpha antibody (1:1,000, Santa Cruz Biotechnology; Dallas, TX, USA). For loading normalization we used anti-β-actin antibody (1:10,000, Sigma-Aldrich). Anti-rabbit (1:1,000, Bio-Rad; Hercules, CA, USA) and anti-mouse (1:5,000, Bio-Rad) antibodies conjugated with horseradish peroxidase were used as secondary antibodies. Bands were visualized using the ECL Western blotting detection system. Images were captured with a Fuji LAS-3000 Imaging System, as previously described [34].

### Real-time quantitative PCR (qPCR)

Total RNA from cells was isolated using the RNeasy Mini Kit (Qiagen; Valencia, CA, USA). One μg of total RNA was reverse transcribed to cDNA with the TaqMan Reverse Transcription Kit (Invitrogen; Carlsbad, CA, USA). Real-time quantitative PCR (qPCR) was performed in triplicate in a total volume of 25 μl containing 10 to 100 ng cDNA, TaqMan universal PCR MasterMix (Applied Biosystems; Branchburg, NJ, USA), target primers (45 nM), and fluorescent probe (12.5 nM), using an ABI PRISM 7700 detection system. TaqMan probes specific for *PREX1*, *VAV3*, *ESR1* and the housekeeping genes *B2M* and *UBC* (used for normalization) were obtained from Applied Biosystems. PCR product formation was continuously monitored using the Sequence Detection System software version 1.7 (Applied Biosystems).

### Analysis of CpG islands in the PREX1 and VAV3 gene promoters

The presence of CpG islands in the human *PREX1* (NM_020820) and *VAV3* (NM_006113) gene promoters was determined using the Methyl Primer Express software (Applied Biosystems).

### Bisulfite sequencing and pyrosequencing

Genomic DNA was isolated from cell lines in culture using the QIAamp DNA Mini Kit (Qiagen). For bisulfite sequencing, 1 μg of genomic DNA was treated with sodium bisulfite and hydroquinone [35] and purified using the Wizard DNA Clean-up system (Promega; Madison, WI, USA). Bisulfite treated-genomic DNA was used as a template to amplify specific promoter regions of *PREX1* and *VAV3*, using the Go-Taq Hot Start Polymerase (Promega). Primer pairs in bisulfite-sequencing PCRs were as follows: 5′-GGAGGATTTTGGAGTTAGGTAT (*PREX1*-BSP1-Forward), 5′-AACAAATACCCTACCTACTCCC (*PREX1*-BSP1-Reverse), 5′-TTAGGGGGTAAAGAAGTTTAGA (*PREX1*-BSP2-Forward), 5′-AACCAAATAAACACC^A^/_G_AACT (*PREX1*-BSP2-Reverse), 5′-GTTAGAATGGAGG^C^/_T_GTTTAG (*PREX1*-BSP3-Forward), 5′-AAAACTATCCCCAAACTCC (*PREX1*-BSP3-Reverse), 5′-GGGATT^C^/_T_GAGTTTTTTTAGA (*PREX1*-BSP4-Forward), 5′-ACTCCAACAAAAACCTATACAT (*PREX1*-BSP4-Reverse), 5′-TTTAAGTAGGTTTTTGTGGGGT (*VAV3*-BSP-Forward) and 5′-CAAACTCCCCAAAACAATAAA (*VAV3*-BSP-Reverse). The thermal cycle conditions consisted of 95°C for 5 min, followed by 35 cycles of denaturation at 95°C for 30 sec, annealing at 57°C for 30 sec, and elongation at 72°C for 1 min, and then an incubation at 72°C for 10 min. PCR products were cloned into the TopoTA vector (Invitrogen). Eight clones for each PCR reaction were randomly selected and sequenced.

For pyrosequencing, bisulfite treated-genomic DNA obtained from cell lines in culture was amplified using the Hs_PREX1_04_PM PyroMark CpG assay (Qiagen) specific for the human *PREX1* promoter, and the PyroMark PCR Kit (Qiagen), using the conditions described by the manufacturer. Pyrosequencing was performed in the Genetic Resources Core Facility at Johns Hopkins University.

### 5-aza-2′-deoxycitidine and trichostatin A treatment

Cells growing in complete media were treated with 10 μΜ 5-aza-2′-deoxycitidine (AZA) (Sigma-Aldrich) for 96 h and/or 100 ng/ml trichostatin A (TSA) (Sigma-Aldrich) for 24 h. At the end of treatment, P-REX1 mRNA expression was determined by qPCR as described above.

### Microarray analysis

For *PREX1* expression and methylation profiles, processed cell line expression data was downloaded from ArrayExpress under the accession number E-TAMB-157 [36]. Processed cell line methylation data was obtained from the Gene Expression Omnibus (GEO) database under the accession number GSE42944 [37]. Gene expression and Illumina 450 k Human Methylation array data for The Cancer Genome Atlas (TCGA) breast cancer samples were obtained from the TCGA data portal. Expression data from MCF-7 cells with ER-alpha depletion was obtained from the GEO database under the accession number GSE27473 [37]. Statistical analyses for microarrays were performed using the R Biostatistical Program [38] with annotation packages installed from Bioconductor [39].

Clinical annotations for the TCGA datasets were obtained from the TCGA data portal. Samples in which ER status was indeterminate were excluded from the ER analysis. HER2 amplification status was given as two variables: FISH and IHC. Equivocal and indeterminate calls were excluded and when discordance occurred between both calls, priority was given to the FISH assay.

### Transfections and luciferase reporter gene assays

For luciferase reporter assays, cells in 12-well plates were co-transfected with 1 μg of pGL3-*PREX1* promoter constructs [40] or empty vector, and 0.1 ng of the *Renilla* reporter vector pRL-TK (Promega), using the transfection reagent X-tremeGENE HP (Roche; Indianapolis, IN, USA). Forty-eight hours after transfection, cells were lysed with passive lysis buffer (Promega), and luciferase activity determined in cell extracts using the Dual-Luciferase Reporter Assay System (Promega).

For transient depletion of ER-alpha, we used ESR1 ON-TARGETplus SMARTpool, (Catalog # L-003401-00-0005), from Dharmacon (Lafayette, CO, USA). ON-TARGETplus Non-Targeting pool (Catalog # D-001810-0-05) was used as a control. RNAi was transfected using Lipofectamine RNAiMax (Invitrogen) following instructions from the manufacturer.

### Statistical analysis

One-way ANOVA test with Bonferroni corrections was applied, with *P* <0.05 considered as statistically significant. Statistical significance for gene expression and probe methylation from cell line and TCGA datasets were assessed using the Mann-Whitney test with Bonferroni correction across groups. Bonferroni correction was also applied when a given gene was analyzed with more than one probe. For these tests, *P* <0.05 was considered as statistically significant. Pairwise correlations were calculated using the Spearman’s ranked correlation test, with *P* <0.05 considered as statistically significant. For the Kaplan-Meier curves, groups of low and high methylation were defined via bifurcation using the median beta value of the specific probe. Statistical difference between survival curves was calculated using the log-rank test.

## Results

### Differential methylation of the PREXgene in breast cancer subtypes

**Figure 1:**
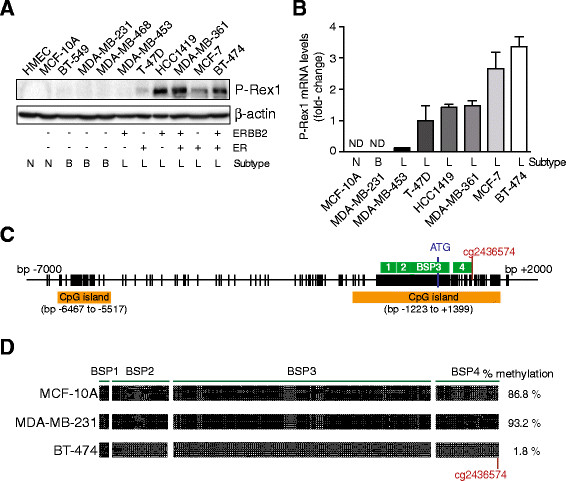
**Differential P-REX1 expression and methylation of the** ***PREX1*** **gene promoter in mammary cell lines.** ***Panel A***. P-REX1 levels in human breast cell lines were determined by Western blot. β-actin was used as loading control. *N*, normal; *B*, basal-like; *L*, luminal. ***Panel B***. Quantitative PCR determination of P-REX1 mRNA levels in human breast cancer cell lines, normalized to those in T-47D cells. *B2M* and *UBC* were used as housekeeping genes. Data are expressed as mean ± S.D. of triplicate samples. A second additional experiment gave similar results. As P-REX1 mRNA levels in MCF-10A are non detectable (*ND*), statistical analysis is not provided. ***Panel C***. Schematic representation of the *PREX1* gene promoter. The location of the two CpG islands, the four regions amplified by BSPs, and the ATG codon are indicated. ***Panel D***. DNA methylation of the *PREX1* promoter was determined by bisulfite sequencing PCR. Each dot represents the methylation status of a CpG dinucleotide in one sequenced BSP clone. *Black dot*, methylated CpG; *white dot*, unmethylated CpG.

We have previously established that the Rac-GEF P-REX1 is overexpressed in breast cancer relative to normal breast tissue, and a positive correlation was found with ER-positive breast tumors [24]. Analysis of databases from human breast specimens revealed elevated P-REX1 mRNA levels mainly in luminal A and luminal B subtypes, whereas expression in the basal-like breast cancer subtype was very low [21],[24]. To extend these results to cellular models, we analyzed P-REX1 expression in a number of human breast cancer cell lines. Western blot analysis showed elevated P-REX1 protein levels in most luminal breast cancer cell lines examined (T-47D, HCC1419, MDA-MB-361, MCF-7, BT-474). On the other hand, P-REX1 was essentially undetectable in basal-like cell lines (BT-549, MDA-MB-231, MDA-MB-468) or in normal mammary epithelial cells (HMEC, MCF-10A) (Figure 1A). This distinctive pattern of expression could also be detected at the mRNA level, as determined by qPCR (Figure 1B).

To dissect the mechanisms behind the differential expression of P-REX1 in breast cancer, we turned our attention to gene promoter methylation. DNA methylation of CpG dinucleotides is an epigenetic alteration that induces transcriptional silencing, and its dysregulation has profound roles on the expression of genes linked to oncogenesis and tumor progression [31]. Analysis of the *PREX1* gene promoter sequence using the Methyl Primer Express software revealed two major CpG islands, defined as sequences covering more than 200 bp with a C + G content over 50% and an observed-to-expected CpG ratio >0.6 [41]. The first CpG island is located 6.5 to 5.5 kb upstream from the ATG start codon. The second (and largest) CpG island is located between -1.2 kb and +1.4 kb, and it includes the first exon of the *PREX1* gene. This proximal CpG island is particularly rich in C + G bases and has an observed-to-expected CpG ratio >0.75 (Figure 1C).

In order to determine the methylation status of the *PREX1* promoter region, we utilized a bisulfite genomic sequencing approach, using three representative breast cell lines for normal (MCF-10A), basal-like (MDA-MB-231), and luminal (BT-474) subtypes. Four different bisulfite sequencing PCRs (BSPs) were designed in order to cover most of the proximal CpG island. This analysis revealed prominent hypermethylation of the *PREX1* promoter in MCF-10A cells (86.8% CpG dinucleotides methylated) and MDA-MB-231 cells (93.2%). On the other hand, this CpG island was essentially unmethylated in BT-474 cells (1.8%) (Figure 1D). Thus, the methylation status of the *PREX1* promoter inversely correlates with P-REX1 mRNA and protein levels in MCF-10A, MDA-MB-231 and BT-474 cells.

**Figure 2:**
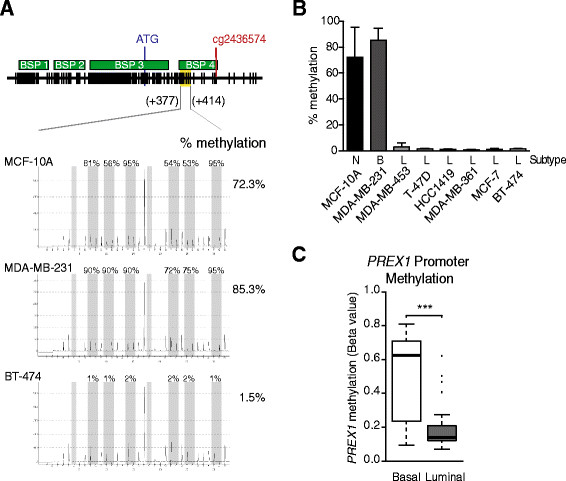
**DNA methylation status of the** ***PREX1*** **promoter in human mammary cell lines.** ***Panel A***. Schematic representation of the *PREX1* promoter region studied with the Hs_PREX1_04_PM PyroMark CpG assay (Qiagen), and representative pyrosequencing experiments for MCF-10A, MDA-MB-231, and BT-474 cell lines. ***Panel B***. Pyrosequencing results of the *PREX1* methylation in breast cancer cell lines. Results are expressed as mean ± S.D. of the percentage of methylation for the six CpG dinucleotides measured by the pyrosequencing assay. ***Panel C*** *.* The *PREX1* promoter methylation levels for probe cg24364574 across 51 cancer cell lines from the Infinium HumanMethylation27 BeadChip array (GEO accession number: GSE42944). (***, *P* <0.0001).

To further establish a relationship between *PREX1* gene methylation and P-REX1 expression in cell lines, we determined DNA methylation levels by pyrosequencing, a fully quantitative methylation assessment. Methylation was determined using a PyroMark CpG assay in a region between +377 bp and +414 bp in the *PREX1* promoter that includes six CpG dinucleotides. In agreement with results from the BSP analysis, pyrosequencing revealed high methylation in normal MCF-10A and basal-like MDA-MB-231 cells (72.3% and 85.3% methylation, respectively). On the other hand, methylation was essentially undetectable in luminal cell lines (<3% in all cell lines examined) (Figure 2A and 2B), which in all cases display high P-REX1 mRNA levels by qPCR (see Figure 1B).

Next, we analyzed the methylation status of *PREX1* in 51 breast cancer cell lines obtained from the Infinium HumanMethylation27 BeadChip array (Illumina; San Diego, CA, USA); GSE42944 [37]). This array contains data on cg24364574, a CpG dinucleotide located at bp +703 in the *PREX1* gene promoter. Again, this analysis revealed major differences between basal-like cell lines that display high methylation on cg24364574 and luminal-derived cell lines (Figure 2C; *P* <0.001). Taken together, analysis of human mammary cell lines argue for a major role of methylation in repressing the expression of P-REX1 in normal breast tissue and basal-like breast tumors. It is conceivable that derepression of P-REX1 expression in luminal breast cancer is associated with demethylation of the *PREX1* promoter.

### Rescue of P-REX1 expression in normal mammary and basal-like breast cancer cells by 5-aza-2′-deoxycytidine

**Figure 3:**
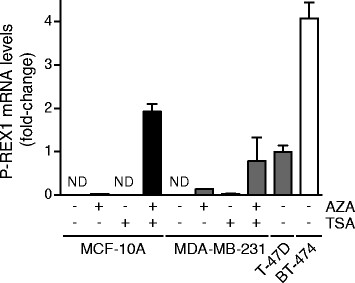
**AZA restores the expression of P-REX1 in MCF-10A and MDA-MB-231 cells.** Cells were treated with 5-aza-2′-deoxycytidine (AZA) (10 μM, 96 h) and/or trichostatin A (TSA) (24 h, 100 ng/ml). Total RNA was isolated, reverse transcribed, and used for the determination of P-REX1 mRNA levels by quantitative PCR. T-47D and BT-474 breast cancer cell lines were used as positive controls for P-REX1 expression in luminal breast cancer cell lines. P-REX1 mRNA levels were normalized to the housekeeping gene *B2M* and expressed as relative to those in T-47D cells. Similar results were observed in two additional experiments. As P-REX1 mRNA levels in non-treated MCF-10A and MDA-MB-231 cells are non detectable (*ND*), statistical analysis is not provided.

To determine if P-REX1 expression is regulated by methylation, we used the demethylating agent 5-aza-2′-deoxycytidine (AZA). As histone deacetylation usually works together with DNA methylation in the silencing of genes [42],[43], AZA treatment was done either in the presence or absence of the histone deacetylase (HDAC) inhibitor trichostatin A (TSA). As shown in Figure 3, combined AZA/TSA treatment rescued P-REX1 expression in MCF-10A and MDA-MB-231 cells to levels in the range of those observed in T-47D or BT-474 cells. P-REX1 expression could not be rescued by treatment with AZA or TSA alone. These results suggest that the *PREX1* promoter is inactivated by DNA methylation in non-expressing cell lines, and that demethylation in combination with acetylation of associated histones is sufficient to rescue P-REX1 expression.

### Analysis of PREXmRNA expression and promoter methylation in human breast specimens from the TCGA database revealed presence of major differences in breast cancer subtypes

**Figure 4:**
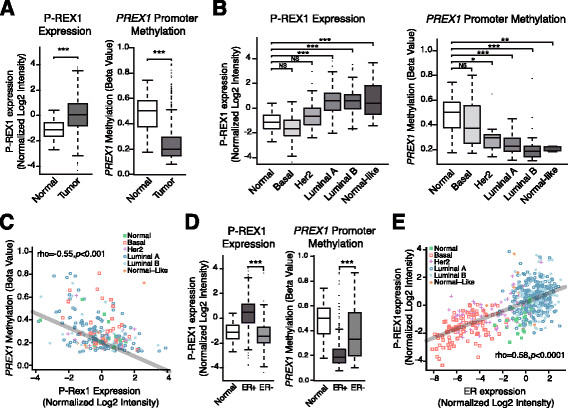
**Analysis of** ***PREX1*** **methylation and mRNA expression in the TCGA dataset.** ***Panel A*** *.* P-REX1 mRNA expression (*left*) and promoter methylation (*right*) in breast tumors and normal breast tissue (*** = *P* <0.0001). ***Panel B*** *.* P-REX1 mRNA expression (*left*) and promoter methylation (*right*) across different breast cancer subtypes (*, *P* <0.05; **, *P* <0.01; ***, *P* <0.001). ***Panel C*** *.* Inverse correlation between P-REX1 mRNA levels and *PREX1* methylation in different subtypes of breast tumors and normal breast tissue (Spearman rho = -0.55, *P* <0.0001). ***Panel D*** *.* P-REX1 mRNA expression (*left*) and promoter methylation (*right*) in normal tissue, estrogen receptor (ER)-positive and ER-negative breast tumors (***, *P* <0.0001). ***Panel E*** *.* Correlation analysis between P-REX1 and ER-alpha expression in different subtypes of human breast tumors and normal breast samples (Spearman rho =0.85, *P* <0.0001). TCGA, The Cancer Genome Atlas.

Following the studies in mammary cell lines, we next examined *PREX1* gene methylation in human breast cancer specimens using the TCGA public database. In agreement with IHC data [24], this database also showed significant P-REX1 mRNA upregulation in primary breast carcinomas *vs.* normal breast tissue samples (Figure 4A, *left panel*, *P* <0.001). Analysis of cg24364574 methylation status in the TCGA database revealed significantly lower methylation in primary breast tumors relative to normal breast tissue (Figure 4A, *right panel*, *P* <0.001).

The TCGA database showed significantly higher levels of P-REX1 in luminal A and B breast cancer relative to other cancer subtypes and normal tissues (Figure 4B, *P* <0.001). Analysis of cg24364574 methylation status in the TCGA database revealed remarkable differences among the different breast cancer subtypes. Specifically, high methylation was observed in both basal-like tumors and normal breast tissue, whereas a profound hypomethylation in *PREX1* was detected in those subtypes with high P-REX1 expression, namely luminal A/B and normal-like subclasses (Figure 4B, *P* <0.01). The HER2/ErbB2 subtype displays slightly higher levels of methylation compared to those of luminal subtype. Figure 4C shows a clear inverse correlation between P-REX1 expression levels and *PREX1* gene promoter methylation status in human breast cancer and normal breast specimens (rho = -0.55; *P* <0.001).

Further analysis using the TCGA database revealed *PREX1* hypomethylation in ER-positive tumors relative to ER-negative breast tumors (Figure 4D, *left panel*, *P* <0.001), which inversely correlates with P-REX1 mRNA expression (Figure 4D, *right panel*, *P* <0.001). In fact, P-REX1 levels in breast tumors positively correlates with the expression of the ER-alpha gene, *ESR1* (Figure 4E, rho =0.58; *P* <0.0001). Interestingly, analysis from the GSE27473 database revealed significant reduction in P-REX1 mRNA expression in MCF-7 cells upon ER-alpha short hairpin (sh)RNA depletion. We validated these results experimentally both in MCF-7 and T-47D cells (Figure S1 in Additional file 1). Our analysis did not reveal statistically significant changes in *PREX1* methylation when we compared HER2/ErbB2-positive and HER2/ErbB2-negative tumors (Figure S2 in Additional file 2).

### Demethylation of the PREXpromoter gene is associated with reduced breast cancer survival

**Figure 5:**
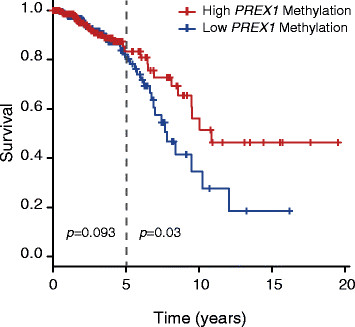
***PREX1*** **methylation predicts poor survival of breast cancer patients.** Kaplan-Meier curve and log-rank test for the survival of breast cancer patients with low or high *PREX1* methylation levels was obtained from the TCGA database. Two *P* values were calculated, one for the comparison of the two groups since time 0 (*P* =0.093), and a second one after year 5 (*P* =0.03). TCGA, The Cancer Genome Atlas.

Montero *et al.* reported a correlation between P-REX1 expression and poor patient outcome in breast cancer [25]. In addition, we previously observed that expression of P-REX1 in primary breast tumors is statistically significantly associated with the development of metastasis [24]. We therefore asked if methylation of the *PREX1* promoter is linked to survival in patients with breast cancer. As shown in Figure 5, low *PREX1* methylation associates with elevated risk of breast cancer mortality. This association becomes statistically significant when we compare patient survival five years after initial diagnosis (*P* =0.03), suggesting a notable trend of poor long-term survival in patients with lower *PREX1* methylation.

### VAVexpression in breast cancer is not regulated by methylation

**Figure 6:**
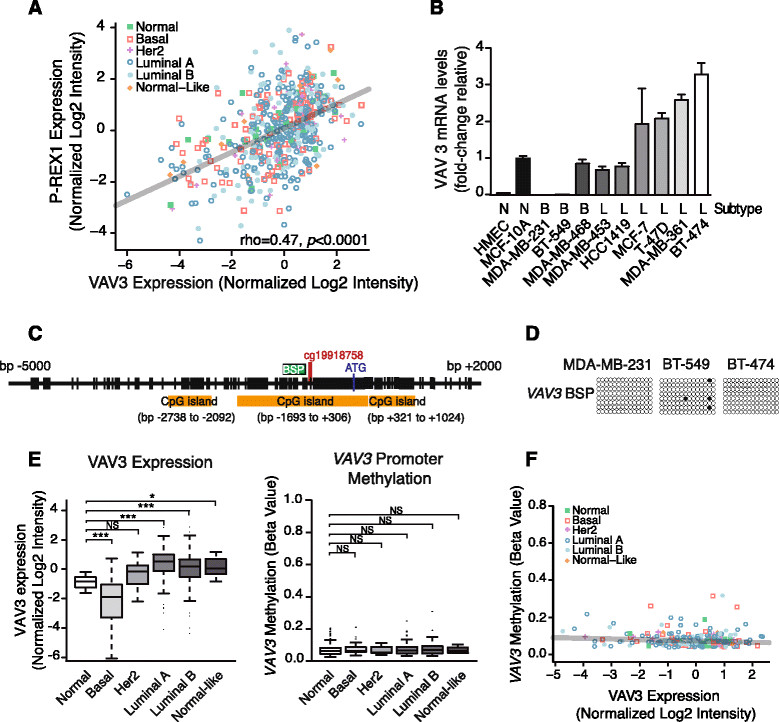
**VAV3 expression is not regulated by promoter methylation.** ***Panel A*** *.* Correlation analysis for P-REX1 and VAV3 expression in breast tumors and normal breast tissue was obtained from the TCGA database (Spearman rho =0.47, *P* <0.0001). ***Panel B*** *.* Quantitative PCR determination of VAV3 mRNA levels in human breast cancer cell lines, normalized to those in immortalized normal breast epithelial cells, MCF-10A cells. Data are expressed as mean ± S.D. of triplicate samples. *B2M* and *UBC* were used as housekeeping genes. A second additional experiment gave similar results. ***Panel C*** *.* Schematic representation of the *VAV3* gene promoter. The location of the three CpG islands, the region amplified by BSP, and the ATG codon are indicated. ***Panel D*** *.* DNA methylation of the *VAV3* promoter was determined by bisulfite sequencing PCR. Each dot represents the methylation status of a CpG dinucleotide in one sequenced BSP clone. *Black dot*, methylated CpG; *white dot*, unmethylated CpG. ***Panel E*** *. VAV3* mRNA expression (*left*) and promoter methylation (*right*) across different breast cancer subtypes and normal mammary tissue were obtained from the TCGA database (*, *P* <0.05; ***, *P* <0.001). ***Panel F*** *.* Correlation analysis for VAV3 mRNA expression and *VAV3* promoter methylation. No significant correlation was observed (Spearman rho = -0.10, *P* =0.14). TCGA, The Cancer Genome Atlas.

Citterio *et al.* recently reported that other Rac-GEFs in addition to P-REX1 are upregulated in luminal breast cancer. Specifically, VAV3 is overexpressed in luminal breast cancer cells, and this drives a transcriptional program for metastatic dissemination to the lungs. Moreover, VAV3 upregulation correlates with ER and PR expression in breast cancer cells [21]. Concordant with these data, we found a strong correlation between P-REX1 and VAV3 expression in human breast samples (Figure 6A; rho =0.47; *P* <0.0001). VAV3 mRNA levels tend to be higher in luminal-derived breast cancer cell lines than in basal-like cancer cell lines or normal mammary cells (Figure 6B). We then asked if VAV3 expression is regulated by methylation. The Methyl Primer Express software identified three CpG islands in the *VAV3* gene promoter (Figure 6C). For the analysis of the methylation status of the *VAV3* promoter we used bisulfite sequencing. Cell lines with low VAV3 expression (MDA-MB-231 and BT-549, basal-like) and high VAV3 expression (BT-474, luminal) were selected. As shown in Figure 6D, sequencing of the BSP region comprising bp -989 to -643 revealed essentially no CpG island methylation in these cell lines. Using the TCGA database, we analyzed the methylation status of the CpG dinucleotide cg19918758 located at bp -623 in the *VAV3* promoter. We found low methylation across all samples and no significant differences in the pattern of methylation between the different breast cancer subtypes (Figure 6E). Additionally, there is no statistically significant correlation between *VAV3* promoter methylation and VAV3 mRNA expression (Figure 6F; rho = -0.10; *P* =0.13). Therefore, methylation plays a major role in controlling P-REX1 expression in breast cancer; however, it does not seem to be a primary mechanism for the control of VAV3 expression.

## Discussion

Rac plays important roles in breast cancer cell migration and invasiveness and is upregulated in invasive human breast cancer [7]-[9],[11]-[13]. Previous studies identified the Rac-GEF P-REX1 as a mediator of Rac1 activation and motility of breast cancer cells in response to growth factors and chemokines [24],[25],[44]. P-REX1 is predominantly upregulated in luminal A and B breast cancer, and its expression is higher in primary breast tumors from patients that ultimately develop metastasis. P-REX1-positive cells can be readily detected in the lymph nodes of patients with breast cancer, strongly arguing for the involvement of this Rac-GEF in the local metastatic dissemination of breast cancer cells [24]. In addition to its involvement in breast cancer, P-REX1 also confers an invasive phenotype to prostate cancer and melanoma cells [26],[27]. The main goal of this study was to decipher the mechanisms behind P-REX1 upregulation in luminal breast cancer, which remained unexplored to date. Our results identified methylation of the *PREX1* gene promoter as a key mechanism implicated in the differential expression of P-REX1 in breast cancer. On the other hand, methylation does not seem to be involved in the upregulation of VAV3, a Rho/Rac-GEF also implicated in breast cancer metastasis [21], despite the presence of CpG islands in the *VAV3* gene promoter. VAV3 is basally expressed in all different breast cancer subtypes despite a preferential overexpression in luminal breast cancer, thus suggesting that mechanisms other than promoter methylation regulate its differential expression.

The *PREX1* promoter has two CpG islands, one of them very rich in C + G bases located between -1.2 kb and +1.4 kb. Our results indicate a differential methylation pattern of this CpG island between different breast cancer subtypes. A systematic analysis of human breast cancer cell lines and tumors revealed that this promoter region is highly methylated in the normal breast epithelium and basal-like breast cancer, and hypomethylated in luminal breast cancer. The inverse correlation found between *PREX1* promoter methylation and P-REX1 expression strongly indicates a role for this epigenetic mechanism in regulating the P-REX1-Rac signaling pathway in breast cancer cells through the control of P-REX1 expression. Additionally, P-REX1 expression can be induced by treatment with the demethylating agent AZA in combination with the HDAC inhibitor TSA in breast cell lines with *PREX1* promoter hypermethylation. Regardless of few studies suggesting indirect effects of these agents [45],[46], our results argue for a distinctive demethylation of the *PREX1* promoter that contributes to its upregulation in the luminal subtype. Interestingly, an association between *PREX1* promoter hypomethylation and reduced overall survival in patients was observed, which reaches statistical significance when considering patients five years after diagnosis. It should be noted that a previous reported analysis of clinical data indicates that patients with elevated P-REX1 levels in breast tumors had a shorter disease-free survival, and a multivariate analysis for known prognostic markers in breast cancer showed that P-REX1 is an independent marker [25]. Taken together, these findings support the concept that the methylation status of the *PREX1* promoter is causally linked to P-REX1 upregulation and associates with poor outcome of breast cancer patients, thus implying *PREX1* methylation as a prognostic factor. Although basal-like tumors have the worse prognosis particularly within the first five years after diagnosis [47], most breast cancers belong to the luminal type. Therefore, survival correlations with P-REX1 expression most likely include luminal breast cancer patients as the largest population, thus arguing the possibility that *PREX1* promoter methylation has prognostic value to predict outcome in this subset of patients. It is important to note that studies using three-dimensional organotypic cultures showed that Rac activity is important for tumor invasion regardless of subtype [13]. This suggests that Rac-GEFs other than P-REX1 are implicated in Rac activation in basal-like tumors.

DNA methylation is a primary epigenetic mechanism for the silencing of genes that has been widely associated with all stages of cancer development, and specific methylation events have been used as biomarkers for diagnosis and prognosis [48],[49]. One well-established alteration linked to cancer development is the inactivation of tumor suppressor genes by DNA methylation. For example, epigenetic silencing by methylation of *PTEN* and *BRCA1* genes is a hallmark of breast cancer [50],[51]. Predictably, inhibition of DNA methylation has been extensively considered as a therapeutic approach, and DNA methylation inhibitors have been approved for cancer therapy [52]. Notwithstanding, fewer studies have addressed a role for abnormal demethylation in cancer, although hypomethylation of the genome has been increasingly recognized as a cancer-linked trait, including in breast cancer [53],[54]. For example, early studies found that hypomethylation of cancer-linked satellite 2 (Sat2) in chromosome 1 is significantly associated with ovarian and breast cancer [53],[55]. Subsequent studies showed that the expression of oncogenic proteins could be activated by abnormal hypomethylation, as shown for *MYC*, *H-RAS*, *R-RAS*, *BCL2* and *PIK3CA*[56]-[61]. Landscapes of promoter demethylation and common hypomethylation signatures have also been established for various cancers [62]-[65]. In addition, DNA demethylation occurs during the process of malignant cell transformation by oncogenes and carcinogenic agents [66]-[68], and reduced methylation in normal tissue was shown to predict predisposition to multiple cancers [69]. Most remarkably, global expression analysis identified hypomethylation of pathways critical for growth and metastasis in cancer [62],[70],[71]. Indeed, hypomethylation of gene promoters for invasive/metastatic proteins such as uPA and MMP2 has been reported [72]-[74]. There is so far little evidence that components of the Rac pathway, a cascade that plays fundamental roles in motility and invasiveness, could be regulated at the epigenetic level. One study showed that expression of the metastatic exchange factor Tiam1 in colon cancer is inversely related to the methylation status of its promoter; however, *TIAM1* gene hypermethylation also occurs in many tumors, and a clear relationship with metastasis could not be observed [75]. Wong *et al.* showed that P-REX1 expression in prostate epithelial cells could be stimulated by a HDAC inhibitor, and suggested that disassociation of HDACs from the transcription factor Sp1 on the *PREX1* promoter may contribute to aberrant P-REX1 upregulation in metastatic prostate cancer [40]. We found similar results in MCF-7, BT-474, and HCC1419 breast cancer cells (a representative experiment in MCF-7 cells is shown in Figure S3 in Additional file 3). It has been reported that methylation of adjacent CpG sites in the Sp1 DNA consensus sequence can affect Sp1 binding [76]. However, we found that in breast cancer cells the luciferase activity of a *PREX1* promoter reporter that includes the Sp1 sites (located in the proximal CpG island, positions -201/-192 and -170/-161) does not change after methylation (data not shown), suggesting that methylation of the Sp1 sites is not relevant for controlling the expression of the gene. At the present time, we do not know if demethylation of the *PREX1* promoter is a consequence of global aberrant hypomethylation as reported in breast cancer [53] or whether it is dictated by a specific signal. We also found that P-REX1 and ER-alpha expression correlates in breast cancer and that depletion of ER-alpha in ER-positive cells reduces P-REX1 mRNA levels. As ER-alpha RNAi does not seem to significantly change the methylation status of *PREX1* promoter (data not shown), it is possible that ER-alpha controls P-REX1 expression by alternative mechanisms.

## Conclusions

In summary, our study established methylation as a major mechanism that dictates the differential expression of P-REX1 in breast cancer subtypes. Increased expression of P-REX1 in luminal breast cancer is associated with demethylation of CpG islands in the *PREX1* promoter. The P-REX1/Rac pathway plays an important role in ErbB receptor-driven breast cancer cell motility and invasiveness, and consequently the methylation status of the *PREX1* promoter could be a determinant in the progression of subsets of breast cancer patients. In addition to the prognostic implications of our findings, our results may have significant impact for cancer therapy. Indeed, the use of demethylating agents is emerging as a novel approach to cancer therapy due to their ability to reactivate the expression of tumor suppressor genes that are silenced by DNA methylation, and studies have proposed the use of AZA or related agents as anti-cancer agents for patients with solid tumors [77]-[82]. Specifically for breast cancer, demethylating agents have been shown to overcome resistance to other agents such as tamoxifen [83]-[85]. Thus, the fact that tumor-promoting and metastatic genes such as *PREX1* can be reactivated by demethylating agents poses a serious therapeutic challenge, specially taking into account that *PREX1* demethylation is correlated with poor prognosis.

## Authors' contributions

LB-R participated in the study design, performed most of the experimental work, contributed to data analysis and interpretation, drafted the manuscript, and critically revised it. LGB participated in the study design, carried out part of the experimental work, and contributed to data analysis and interpretation. SC made substantial contribution to acquisition, analysis, and interpretation of data, and helped to draft the manuscript. NE, SS and YT participated in the study design, contributed to data analysis and interpretation, helped to draft the manuscript, and revised it critically for intellectual content. MGK conceived and designed the study, interpreted the data, co-drafted, and critically revised the manuscript. All authors read and approved the final manuscript.

## Additional files

## Electronic supplementary material

Additional file 1: Figure S1.: ER-alpha RNAi depletion reduces P-REX1 expression. *Panel* ***A***. P-REX1 mRNA expression in MCF-7 cells subject to estrogen receptor (ER)-alpha depletion, from dataset GSE27473 (***, *P* <0.001). *Panels* ***B***. P-REX1 mRNA levels in MCF-7 (*left panel*) and T-47D cells (*right panel*) were determined after transfection with either ER-alpha or non-target control (*NTC*) RNAi. P-REX1 mRNA levels were normalized to the housekeeping gene *B2M* and expressed as relative to those in NTC-transfected cells. Similar results were observed in an additional experiment. ***, *P* <0.001. *Inset*, ER-alpha expression was determined by Western blot. (PDF 325 KB)

Additional file 2: Figure S2.: Similar *PREX1* expression and methylation across HER2 subtypes. *PREX1* mRNA expression (*left*) and promoter methylation (*right*) in normal tissue, HER2/ErbB2-positive and HER2/ErbB2-negative breast cancers values were obtained from The Cancer Genome Atlas (TCGA) database. No statistically significant differences were observed between HER2/ErbB2-positive and -negative tumors. *NS*, not significant. (PDF 341 KB)

Additional file 3: Figure S3.: Sp1 sites are required for transcriptional activity of the *PREX1* promoter. *Panel* ***A***. Luciferase activity of truncated deletions of the *PREX1* promoter (cloned in a pGL3 vector) was measured in MCF-7 cells. Luciferase activity was determined 48 h after transfection. Data are expressed as mean ± S. D. relative to construct comprising bp -599 to bp -20. *Panel* ***B***. Luciferase assay was performed upon transfection of *PREX1* luciferase constructs comprising bp -204 to bp -20, either wild-type or with both Sp1 sites mutated [40]. Cells were treated either with trichostatin A (TSA) (100 ng/ml, 24 h) or vehicle. Data are expressed as mean ± S. D. relative to wild-type. Experiments were done in triplicate, and similar results were observed in three separate experiments. *** = *P* <0.001. (PDF 335 KB)

Below are the links to the authors’ original submitted files for images.

Authors’ original file for figure 1

Authors’ original file for figure 2

Authors’ original file for figure 3

Authors’ original file for figure 4

Authors’ original file for figure 5

Authors’ original file for figure 6

## Abbreviations

ATCC: American Type Culture Collection
AZA: 5-aza-2-deoxycytidine
B2M: beta-2 microglobulin
BSP: bisulfite sequencing PCR
ER-alpha: estrogen receptor-alpha
GAP: GTPase-activating protein
GEF: guanine-nucleotide exchange factor
GPCR: G protein-coupled receptor
HDAC: histone deacetylase
IHC: immunohistochemistry
ND: non detectable
PCR: polymerase chain reaction
PI3K: phosphoinositide 3-kinase
P-REX1: phosphatidylinositol 3,4,5-trisphosphate-dependent Rac exchanger 1
qPCR: quantitative PCR
Rac1: Ras-related C3 botulinum toxin substrate 1
RNAi: RNA interference
TSA: trichostatin A
UBC: ubiquitin C
