# Supplementary figures and images for: Subtype-specific overexpression of the Rac-GEF P-REX1 in breast cancer is associated with promoter hypomethylation
Source: Breast Cancer Res. 2014 Sep 24;16:441. doi: 10.1186/s13058-014-0441-7 (PMC4303123; doi:10.1186/s13058-014-0441-7)

**A**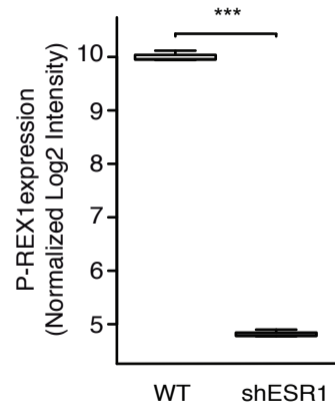**B**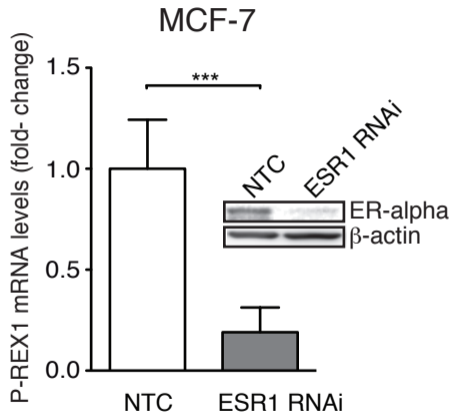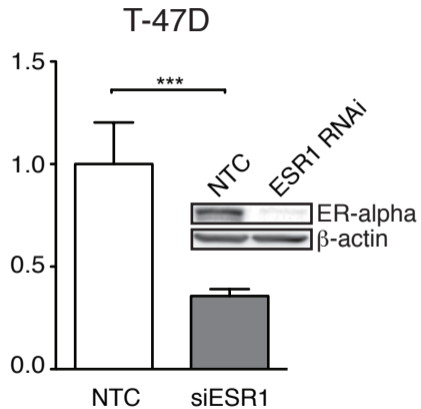

Supplement: Supplementary file 1 — Additional file 1: Figure S1.: ER-alpha RNAi depletion reduces P-REX1 expression. Panel A. P-REX1 mRNA expression in MCF-7 cells subject to estrogen receptor (ER)-alpha depletion, from dataset GSE27473 (***, P <0.001). Panels B. P-REX1 mRNA levels in MCF-7 (left panel) and T-47D cells (right panel) were determined after transfection with either ER-alpha or non-target control (NTC) RNAi. P-REX1 mRNA levels were normalized to the housekeeping gene B2M and expressed as relative to those in NTC-transfected cells. Similar results were observed in an additional experiment. ***, P <0.001. Inset, ER-alpha expression was determined by Western blot. (PDF 325 KB) [file 13058_2014_441_MOESM1_ESM.pdf]

## P-REX1 Expression

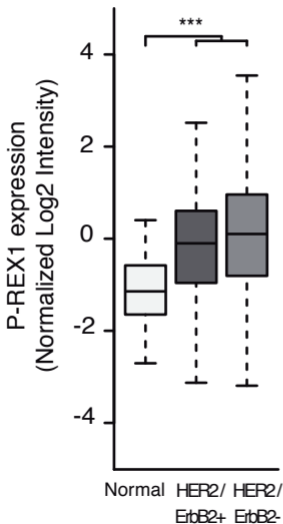

## *PREX1* Promoter Methylation

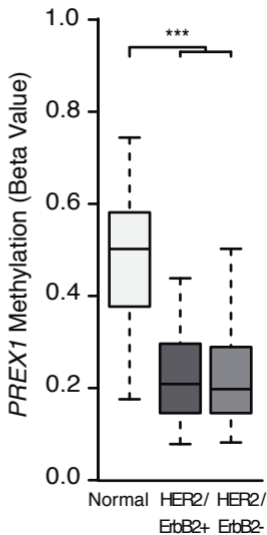

Supplement: Supplementary file 2 — Additional file 2: Figure S2.: Similar PREX1 expression and methylation across HER2 subtypes. PREX1 mRNA expression (left) and promoter methylation (right) in normal tissue, HER2/ErbB2-positive and HER2/ErbB2-negative breast cancers values were obtained from The Cancer Genome Atlas (TCGA) database. No statistically significant differences were observed between HER2/ErbB2-positive and -negative tumors. NS, not significant. (PDF 341 KB) [file 13058_2014_441_MOESM2_ESM.pdf]

**A**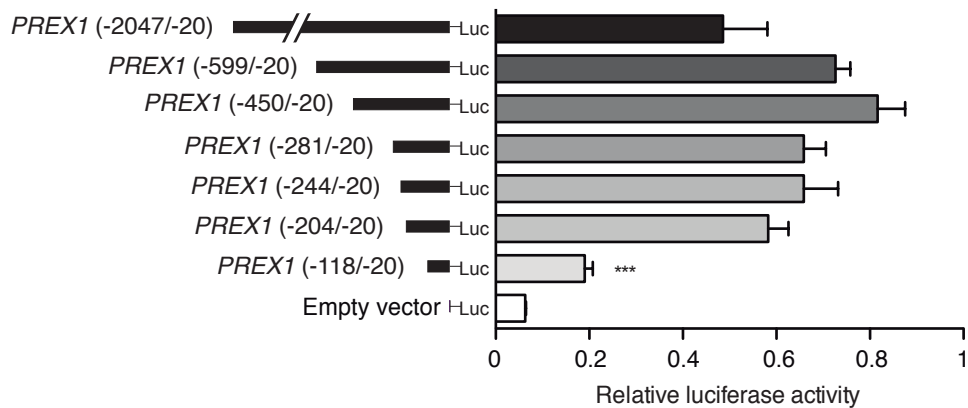**B**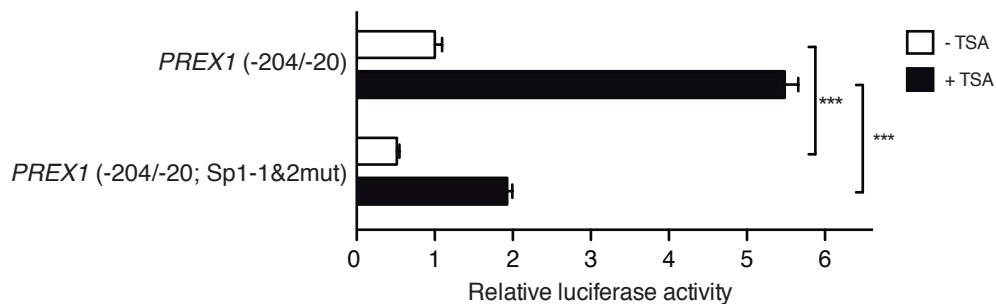

Supplement: Supplementary file 3 — Additional file 3: Figure S3.: Sp1 sites are required for transcriptional activity of the PREX1 promoter. Panel A. Luciferase activity of truncated deletions of the PREX1 promoter (cloned in a pGL3 vector) was measured in MCF-7 cells. Luciferase activity was determined 48 h after transfection. Data are expressed as mean ± S. D. relative to construct comprising bp -599 to bp -20. Panel B. Luciferase assay was performed upon transfection of PREX1 luciferase constructs comprising bp -204 to bp -20, either wild-type or with both Sp1 sites mutated [40]. Cells were treated either with trichostatin A (TSA) (100 ng/ml, 24 h) or vehicle. Data are expressed as mean ± S. D. relative to wild-type. Experiments were done in triplicate, and similar results were observed in three separate experiments. *** = P <0.001. (PDF 335 KB) [file 13058_2014_441_MOESM3_ESM.pdf]

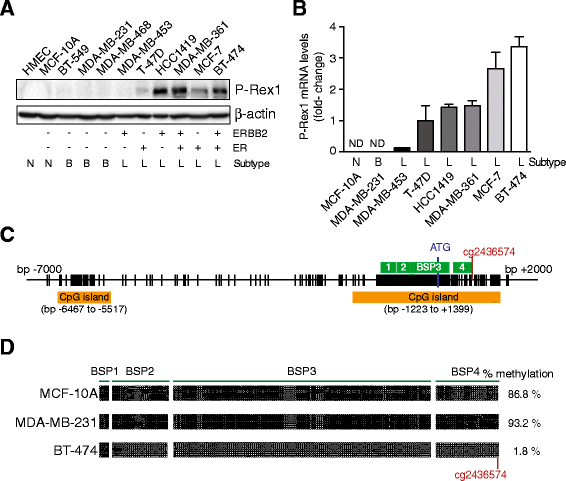

Supplement: Supplementary file 4 — Authors’ original file for figure 1 [file 13058_2014_441_MOESM4_ESM.gif]

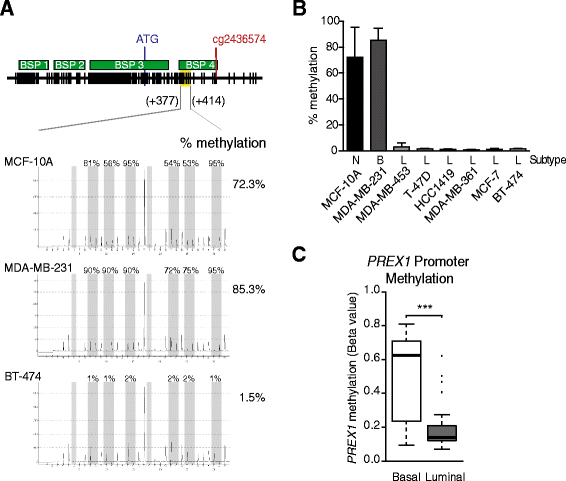

Supplement: Supplementary file 5 — Authors’ original file for figure 2 [file 13058_2014_441_MOESM5_ESM.gif]

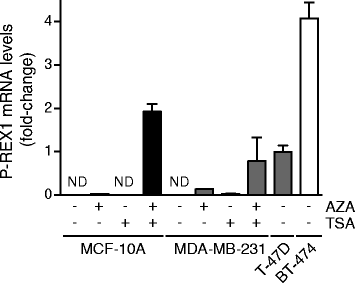

Supplement: Supplementary file 6 — Authors’ original file for figure 3 [file 13058_2014_441_MOESM6_ESM.gif]

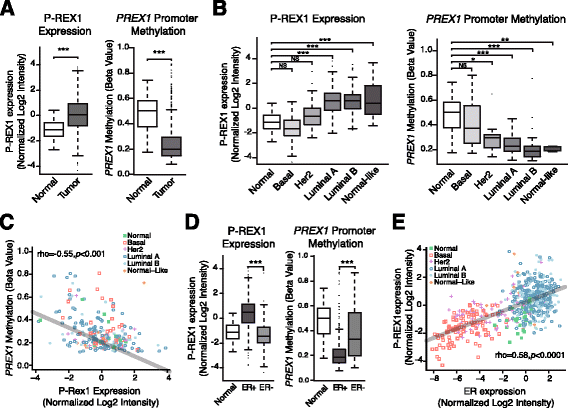

Supplement: Supplementary file 7 — Authors’ original file for figure 4 [file 13058_2014_441_MOESM7_ESM.gif]

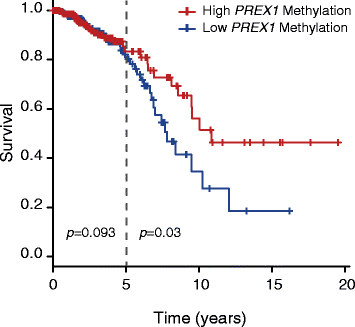

Supplement: Supplementary file 8 — Authors’ original file for figure 5 [file 13058_2014_441_MOESM8_ESM.gif]

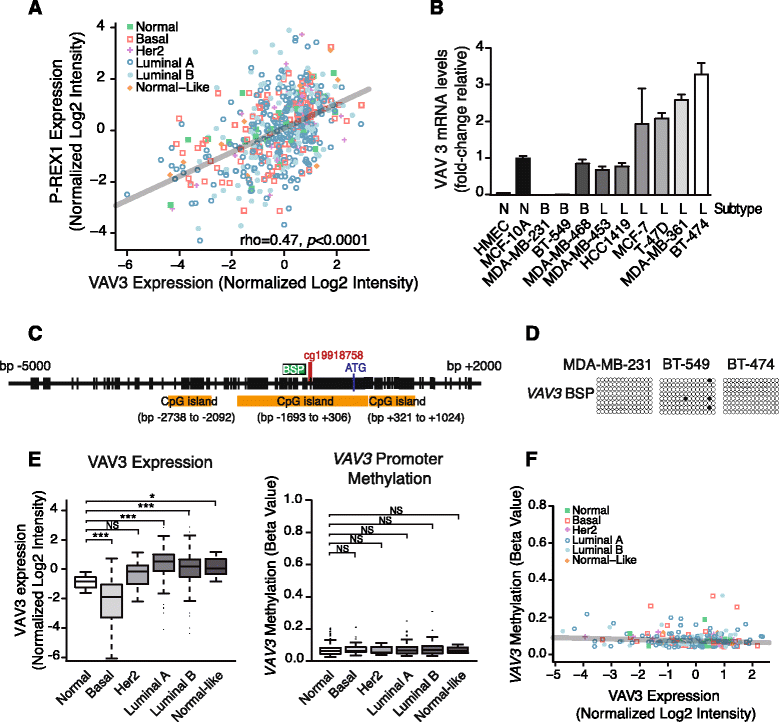

Supplement: Supplementary file 9 — Authors’ original file for figure 6 [file 13058_2014_441_MOESM9_ESM.gif]
